# Astrocytes Modulate Baroreflex Sensitivity at the Level of the Nucleus of the Solitary Tract

**DOI:** 10.1523/JNEUROSCI.1438-19.2020

**Published:** 2020-04-08

**Authors:** Svetlana Mastitskaya, Egor Turovsky, Nephtali Marina, Shefeeq M. Theparambil, Anna Hadjihambi, Sergey Kasparov, Anja G. Teschemacher, Andrew G. Ramage, Alexander V. Gourine, Patrick S. Hosford

**Affiliations:** ^1^Centre for Cardiovascular and Metabolic Neuroscience, Department of Neuroscience, Physiology, and Pharmacology, University College London, London WC1E 6BT, United Kingdom,; ^2^Institute of Cell Biophysics, Federal Research Center “Pushchino Scientific Center for Biological Research of the Russian Academy of Sciences”, Pushchino 142290, Russian Federation,; ^3^Physiology, Pharmacology, and Neuroscience, University of Bristol, Bristol BS8 1TD, United Kingdom,; ^4^Baltic Federal University, Kaliningrad 236041, Russian Federation, and; ^5^William Harvey Research Institute, Barts and The London School of Medicine and Dentistry, Queen Mary University of London, London EC1M 6BQ, United Kingdom

**Keywords:** astrocytes, baroreflex, cardiovascular, *in vivo*, nucleus of the solitary tract, viral gene transfer

## Abstract

Maintenance of cardiorespiratory homeostasis depends on autonomic reflexes controlled by neuronal circuits of the brainstem. The neurophysiology and neuroanatomy of these reflex pathways are well understood, however, the mechanisms and functional significance of autonomic circuit modulation by glial cells remain largely unknown. In the experiments conducted in male laboratory rats we show that astrocytes of the nucleus of the solitary tract (NTS), the brain area that receives and integrates sensory information from the heart and blood vessels, respond to incoming afferent inputs with [Ca^2+^]_i_ elevations. Astroglial [Ca^2+^]_i_ responses are triggered by transmitters released by vagal afferents, glutamate acting at AMPA receptors and 5-HT acting at 5-HT_2A_ receptors. In conscious freely behaving animals blockade of Ca^2+^-dependent vesicular release mechanisms in NTS astrocytes by virally driven expression of a dominant-negative SNARE protein (dnSNARE) increased baroreflex sensitivity by 70% (*p* < 0.001). This effect of compromised astroglial function was specific to the NTS as expression of dnSNARE in astrocytes of the ventrolateral brainstem had no effect. ATP is considered the principle gliotransmitter and is released by vesicular mechanisms blocked by dnSNARE expression. Consistent with this hypothesis, in anesthetized rats, pharmacological activation of P2Y_1_ purinoceptors in the NTS decreased baroreflex gain by 40% (*p* = 0.031), whereas blockade of P2Y_1_ receptors increased baroreflex gain by 57% (*p* = 0.018). These results suggest that glutamate and 5-HT, released by NTS afferent terminals, trigger Ca^2+^-dependent astroglial release of ATP to modulate baroreflex sensitivity via P2Y_1_ receptors. These data add to the growing body of evidence supporting an active role of astrocytes in brain information processing.

**SIGNIFICANCE STATEMENT** Cardiorespiratory reflexes maintain autonomic balance and ensure cardiovascular health. Impaired baroreflex may contribute to the development of cardiovascular disease and serves as a robust predictor of cardiovascular and all-cause mortality. The data obtained in this study suggest that astrocytes are integral components of the brainstem mechanisms that process afferent information and modulate baroreflex sensitivity via the release of ATP. Any condition associated with higher levels of “ambient” ATP in the NTS would be expected to decrease baroreflex gain by the mechanism described here. As ATP is the primary signaling molecule of glial cells (astrocytes, microglia), responding to metabolic stress and inflammatory stimuli, our study suggests a plausible mechanism of how the central component of the baroreflex is affected in pathological conditions.

## Abstract (precis)

Maintenance of cardiorespiratory homeostasis depends on autonomic reflexes controlled by neuronal circuits of the brainstem. The neurophysiology and neuroanatomy of these reflex pathways are well understood, however, the mechanisms and functional significance of autonomic circuit modulation by glial cells remain largely unknown.

## Introduction

Operation of all fundamental reflexes essential for the maintenance of cardiorespiratory homeostasis is controlled by the autonomic circuits located in the lower brainstem. Cardiorespiratory reflexes ensure autonomic balance and maintain cardiovascular health. Impaired operation of these reflexes (the baroreflex in particular) may contribute to the development of cardiovascular disease and serves as a robust predictor of cardiovascular and all-cause mortality (La Rovere et al., 1998, 2001; McCrory et al., 2016). Brainstem autonomic circuits receive sensory information via afferent fibers running within the IXth (glossopharyngeal) and Xth (vagus) cranial nerves. These afferents terminate in the nucleus of the solitary tract (NTS), located in the dorsal aspect of the brainstem, and release glutamate as the principal transmitter at the first central synapse (Talman, 1997; Baude et al., 2009).

Glutamatergic transmission (essential for the processing of afferent information) in the NTS is modulated by other transmitter systems (Sévoz-Couche and Brouillard, 2017), with 5-hydroxytryptamine [serotonin (5-HT)] playing the key role (Ramage and Villalón, 2008; Hosford and Ramage, 2019). The transmitters and receptors involved in signal processing in the NTS have been extensively studied. However, the role of astrocytes in this brain area is less well understood. This is despite a notable abundance and anatomical complexity of the NTS astrocytes (Dallaporta et al., 2010; Sheikhbahaei et al., 2018a) and significant evidence that astrocytes modulate the activities of other CNS circuits, for example, those involved in learning and memory (Han et al., 2012; Navarrete et al., 2012), control of sleep (Halassa et al., 2009), and regulation of breathing (Gourine et al., 2010; Sheikhbahaei et al., 2018b).

Two previous studies suggested a potentially important role of astrocytes in the mechanisms underlying processing of cardiovascular sensory information in the NTS. McDougal and colleagues reported that electrical stimulation of the solitary tract in a brainstem slice preparation activates the NTS astrocytes (shown by an increase in [Ca^2+^]_i_) via the mechanism involving AMPA receptors (McDougal et al., 2011). Lin et al. (2013) demonstrated that ablation of NTS astrocytes (using the ribosomal toxin saporin) impairs baroreflex sensitivity, alters chemo- and von Bezold–Jarisch reflexes, leading to enhanced blood pressure lability and, in some animals, sudden cardiac death. Together, these findings suggest that NTS astrocytes can respond to vagal input and may play an important role in modulation of cardiovascular reflexes. However, ablation of astrocytes removes the structural and metabolic support they provide to neurons and thus could mask the subtleties of their role in modulating transmission and integration of cardiovascular afferent information by the NTS circuits. Therefore, the importance of astroglial signaling for the operation of the cardiovascular reflexes remains unknown.

In the present study we addressed these questions by performing *in vivo* [Ca^2+^]_i_ imaging in NTS astrocytes expressing a genetically encoded Ca^2+^ indicator. Because 5-HT is known to be released in the NTS from vagal afferent terminals (Jeggo et al., 2005; Oskutyte et al., 2009; Hosford et al., 2015), the presence of 5-HT receptors on NTS glia was studied using [Ca_2_]_i_ imaging *in vivo* and the identity of the NTS astroglial 5-HT receptors was determined *in vitro*. Finally, the importance of astroglial signaling mechanisms for the operation of cardiovascular reflexes was determined by blocking Ca^2+^-dependent vesicular release in NTS astrocytes in conscious rats with cardiovascular phenotyping and the assessment of baroreflex sensitivity.

## Materials and Methods

The experiments were performed in Sprague-Dawley rats in accordance with the European Commission Directive 2010/63/EU (European Convention for the Protection of Vertebrate Animals used for Experimental and Other Scientific Purposes) and the United Kingdom Home Office (Scientific Procedures) Act (1986) with project approval from the Institutional Animal Care and Use Committee of the University College London.

### 

#### 

##### In vivo gene transfer.

Young male Sprague-Dawley rats (100–120 g) were anesthetized with a mixture of ketamine (60 mg/kg, i.m.) and medetomidine (250 μg/kg, i.m.) and placed in a stereotaxic frame. NTS astrocytes were targeted bilaterally to express either a genetically encoded Ca^2+^ indicator GCaMP6 (to record activity) or dominant-negative SNARE protein (dnSNARE; to block vesicular exocytosis; Sheikhbahaei et al., 2018b).

Stable GCaMP6 expression along the rostro-caudal extent of the NTS was achieved by placing two microinjections per side [0.25 μl each, speed of injection 0.1 μl/min; coordinates from calamus scriptorius (1) 0.25 mm rostral, 0.5 mm lateral, 0.5 mm ventral and (2) 0.75 mm rostral, 0.5 mm lateral, 0.5 mm ventral] of an adeno-associated viral vector (AAV) to express GCaMP6 under the control of an enhanced glial fibrillary acidic protein (GFAP) promoter (AAV5.GfaABC1D.cytoGCaMP6f.SV40, titer 7 × 10^11^ viral particles/ml; University of Pennsylvania Vector Core).

To block vesicular release mechanisms in NTS astrocytes, two microinjections (0.25 μl each) per side of the adenoviral vector (AVV) with the enhanced GFAP promoter (Liu et al., 2008) were placed in the NTS bilaterally to drive the expression of dnSNARE (AVV.GfaABC1D.dnSNARE.eGFP, titer 7.7 × 10^9^ viral particles/ml). Validation of dnSNARE specificity and efficacy in blocking vesicular release mechanisms in astrocytes was reported previously (Sheikhbahaei et al., 2018b).

To determine whether the effect of compromised astroglial function on baroreflex sensitivity is specific to the NTS, in a separate group of animals astrocytes of the ventrolateral medulla oblongata (VLM) were transduced to express dnSNARE. This brainstem region contains pre-sympathetic and cardiac vagal preganglionic neurons critical for the operation of the baroreflex. Astrocytes within the VLM were targeted bilaterally with two microinjections per side (1 μl each, 0.1 μl/min) of AVV.GfaABC1D.dnSNARE.eGFP using the following coordinates from bregma: 11 and 12 mm caudal, 2 mm lateral, and 8.5 mm ventral. In the control animals, the astrocytes were targeted to express calcium translocating channelrhodopsin variant (CatCh) fused with eGFP (vector: AVV.GfaABC1D.CatCh.eGFP, titer 2.1 × 10^9^ viral particles/ml). Anesthesia was reversed with atipamezole (1 mg/kg). No complications were observed after the surgery and the animals gained weight normally.

##### Anesthetized animal preparation and calcium imaging in NTS astrocytes in vivo.

Imaging experiments were conducted 4 weeks after the viral injections to allow a high and stable level of GCaMP6 expression. Under isoflurane anesthesia (3% in room air), the femoral artery and femoral vein were cannulated for the arterial blood pressure recordings and the delivery of drugs, respectively. After gaining vascular access, anesthesia was transitioned to α-chloralose (initial dose: 100 mg/kg, i.v., maintenance: 30 mg/kg/h, i.v.) and isoflurane was withdrawn. A tracheotomy was performed and the animals were artificially ventilated using a positive pressure ventilator (tidal volume 8–10 ml/kg; frequency ∼60 strokes/min). The body temperature was maintained at 37.0 ± 0.5°C with a servo-controlled heating blanket. The head of the animal was secured in a stereotaxic frame. Arterial blood samples were taken regularly to monitor blood *P*O_2_, *P*CO_2_, and pH (RAPIDLab 348EX, Siemens). Inspired gas composition and/or rate/volume of the ventilation were adjusted to maintain arterial *P*O_2_ within the range: 100–110 mmHg, arterial *P*CO_2_: 35–45 mmHg, arterial pH 7.35–7.45.

To record [Ca^2+^]_i_ responses in NTS astrocytes, the dorsal surface of the brainstem was exposed as described in detail previously (Gourine et al., 2008). [Ca^2+^]_i_ responses in astrocytes evoked by electrical stimulation of the central end of the vagus nerve (5 s stimulation; 5 Hz, 0.8 mA, 10 ms pulse width) were recorded using a Leica fluorescence microscope and MiCAM02 high-resolution camera (SciMedia). To minimize the movement artifacts, recordings were made under neuromuscular blockade with gallamine triethiodide (initial dose: 50 mg/kg, i.v.; maintenance : 10 mg/kg/h, i.v.). Under neuromuscular blockade, an adequate level of anesthesia was ensured by monitoring heart rate and blood pressure for signs of instability. Because acute changes in blood pressure in response to vagal stimulation were associated with drifts in focal plane affecting image acquisition, the arterial blood pressure was clamped by infusion of a nitric oxide synthase inhibitor Nω-Nitro-l-arginine methyl ester (10 mg/kg, i.v.) and ganglion blocker chlorisondamine (1 mg/kg/h, i.v.). Four stimulations of the vagus were applied: two control stimulations followed by stimulations in the presence of increasing doses of 5-HT_2A_ antagonist ketanserin given systemically (100 and 300 μg/kg, i.v.). Recovery periods of 10 min between stimulations were allowed. In a separate set of experiments, stimulations were performed in the absence and presence of an AMPA receptor antagonist CNQX (10 mm in aCSF; applied topically to the dorsal brainstem). Imaging data were collected and analyzed using MiCaM BV_Ana software.

##### Cell culture and calcium imaging in vitro.

Primary astrocyte-enriched neuroglial cultures were prepared from the cortical, hippocampal, cerebellar, and dorsal brainstem tissue of rat pups (P2–P3 of either sex) as described previously (Kasymov et al., 2013). After isolation, the cells were plated on poly-d-lysine-coated coverslips and maintained at 37°C in a humidified atmosphere of 5% CO_2_ and 95% air for a minimum of 12 d before the experiments. Optical measurements of changes in [Ca^2+^]_i_ were performed using an inverted epifluorescence Olympus microscope equipped with a cooled CCD camera (Retiga, QImaging) as described previously (Angelova et al., 2015; Turovsky et al., 2016). We found that from Day 12 the cell cultures contain a negligible number of neurons. This was confirmed at the end of the recordings by application of high potassium solution as described previously (Turovsky et al., 2015). Astrocytes showed no [Ca^2+^]_i_ responses to K^+^-induced depolarization.

Experiments were performed in a custom-made flow-through imaging chamber in a standard HBSS containing 10 mm HEPES. To visualize [Ca^2+^]_i_ responses, astrocytes were loaded with a Ca^2+^ indicator Fura-2 (5 μm; 30 min incubation; Invitrogen). After incubation with the dye, the culture medium was exchanged for fresh HBSS five times before commencing the imaging experiment. The effects of 5-HT or 5-HT receptor agonists on [Ca^2+^]_i_ in individual astrocytes were recorded. Excitation light was provided by a xenon arc lamp with the beam passing through a monochromator at 340 and 380 nm (Cairn Research) and emitted fluorescence at 515 nm was registered. Imaging data were collected and analyzed using Andor software (Andor). All reported data were obtained from at least six separate experiments.

##### Recordings of the arterial blood pressure and heart rate using biotelemetry.

Systemic arterial blood pressure and heart rate in conscious rats transduced to express dnSNARE or control transgene in the NTS and VLM astrocytes were recorded using biotelemetry, as described previously (Machhada et al., 2017). Rats were anesthetized with isoflurane (3% in O_2_), a laparotomy was performed, and a catheter connected to a biotelemetry pressure transducer (model TA11PA-C40, DSI) was advanced rostrally into the abdominal aorta and secured in place with Vetbond (3M). The transmitter was secured to the abdominal wall and the incision was closed by successive suturing of the abdominal muscle and skin layers. For postoperative analgesia, the animals received carprofen (4 mg/kg/d, i.p.) for 2 d and were allowed to recover for at least 7 d. After the recovery period and following recordings of the baseline hemodynamic parameters for 24 h, the animals received microinjections of the AVVs to express dnSNARE or control transgene in the NTS or VLM astrocytes, as described above. Blood pressure was recorded between 7 and 10 d after the injections of the viral vectors when the brainstem expression of the transgenes is fully established (Rajani et al., 2018; Sheikhbahaei et al., 2018b). Animals expressing dnSNARE in the VLM astrocytes were monitored for 24 h period 7 d after the injections of viral vectors as the effect of targeting the NTS astrocytes peaked at this time point.

##### Analysis of the biotelemetry data.

Recordings of the arterial blood pressure were used to calculate the heart rate and spontaneous baroreflex gain (sBRG) for the light and dark periods of the 24 h cycle sBRG was determined from spontaneous changes in systolic blood pressure and pulse interval as described in detail previously (Oosting et al., 1997; Waki et al., 2003).

##### Assessment of BRG in anesthetized animals.

In animals anesthetized with α-chloralose (initial dose: 100 mg/kg, i.v., maintenance: 30 mg/kg/h, i.v.) and instrumented for the recordings of the arterial blood pressure and heart rate, arterial baroreceptors were activated by intravenous bolus injection of norepinephrine (1 μg/kg). Concomitant changes in blood pressure and heart rate were recorded from three consecutive stimulations delivered with intervals of 3 min. BRG was assessed in the absence and presence of P2Y_1_ receptor antagonist MRS 2500 (5 μm) or agonist MRS 2365 (100 μm), applied on the dorsal surface of the brainstem. The BRG was calculated as a ratio of changes in heart rate to that of mean arterial blood pressure (bpm/mmHg) for reflex bradycardia. BRG values were averaged over three measurements made in control conditions and in the presence of either a P2Y_1_ receptor antagonist or agonist.

##### Histology and immunohistochemistry.

At the end of the experiments, the animals were terminally anesthetized with pentobarbitone sodium (200 mg/kg, i.p.) and perfused transcardially with 0.1 m PBS, pH 7.4. The brainstem was removed and fixed for 24 h in 4% paraformaldehyde in PBS at 4°C, followed by cryoprotection in 30% sucrose. Serial transverse sections (30 μm) of the medulla oblongata were cut using a freezing microtome. Immunohistochemistry was performed on free-floating sections by incubation overnight at 4°C with mouse anti-microtubule-associated protein 2 (MAP2; 1:500; Sigma-Aldrich, M1406), rabbit anti-tyrosine hydroxylase (1:100; Sigma-Aldrich, HPA061003) and/or chicken anti-GFP (1:250; Aves Labs, GFP-1020) followed by incubation with secondary antibodies conjugated to the fluorescent probes for 2.5 h at room temperature (each 1:250; Life Science Technologies). Images were obtained with a confocal microscope (Zeiss LSM 900) or epifluorescent microscope (Leica, DMR).

##### Drugs.

5-HT receptor agonists and antagonists were used to determine the type of 5-HT receptors expressed by brainstem astrocytes: 5-HT_2A_ antagonists ketanserin and MDL 100907, 5-HT_2A_ agonist *N*,*N*-dimethyltryptamine (DMT), 5-HT_2B_ agonist BW 723C86, 5-HT_2C_ agonist WAY 161503, 5-HT_3_ antagonist granisetron. Phospholipase C activity was blocked with U73122. MRS 2365 and MRS 2500 were used to activate or block P2Y_1_ receptors, respectively. AMPA receptors were blocked with CNQX. All drugs were purchased from Tocris Bioscience.

##### Data analysis.

Physiological data obtained in the experiments in anesthetized preparations were recorded and analyzed using *Spike2* software (Cambridge Electronic Design). Built-in analysis software tools (Olympus or MiCAM BV_Ana) were used to analyze the results of the imaging experiments. Differences between the experimental groups/treatments were tested for statistical significance by one-way or two-way ANOVA followed by the *post hoc* Tukey–Kramer test, Student's *t* test or Wilcoxon matched-pairs signed rank test, as appropriate. Data are reported as individual values and mean ± SEM. Differences with *p* < 0.05 were considered to be significant.

## Results

### Vagus nerve simulation activates NTS astrocytes *in vivo*

**Figure 1.**
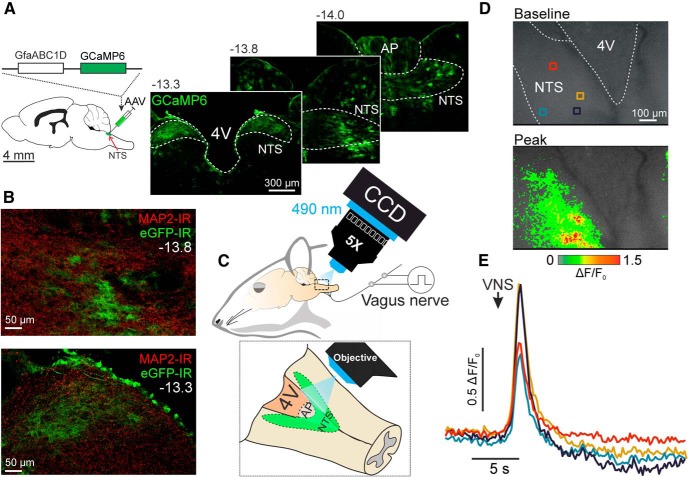
*In vivo* imaging of [Ca^2+^]_i_ responses in astrocytes of the NTS. ***A***, Schematic drawing of the rat brain in sagittal projection illustrating the anatomical location of the NTS targeted to express GCaMP6 in astrocytes under the control of the enhanced GFAP promoter GfaABC1D. GCaMP6 expression in astrocytes of the dorsal vagal complex 4 weeks after the transfection. AP, Area postrema; 4V, fourth ventricle. Distance from bregma (in mm) is indicated. ***B***, Confocal images illustrating NTS astrocytes expressing GCaMP6 (identified by eGFP-immunoreactivity) showing no colocalization of expression with MAP2-immunoreactivity. ***C***, Schematic drawing of the recording setup that included a CCD camera coupled to a low-power microscope to obtain fluorescent images from the dorsal aspect of the brainstem. The central end of the vagus nerve was stimulated electrically. ***D***, False color images of GCaMP6 fluorescence at baseline and at the peak of the response evoked by vagus nerve stimulation (VNS). Colored boxes depict regions-of-interest (ROI). ***E***, Representative changes in GCaMP6 fluorescence in four ROIs (indicated in ***D***) evoked by electrical stimulation of the ipsilateral vagus nerve.

Strong expression of GCaMP6 was observed in astrocytes residing in the mediolateral and rostrocaudal extent of the dorsal vagal complex including the NTS, the area postrema and the dorsal motor nucleus of the vagus nerve (Fig. 1*A*). No colocalization between GCaMP6 expression (visualized by GFP immunoreactivity) and that of a neuronal marker MAP2 (Matus, 1990) was observed, confirming the specificity of astroglial targeting (Fig. 1*B*).

Rapid increases in GCaMP6 fluorescence intensity (0.98 ± 0.24 Δ*F*/*F*_0_; *n* = 5) were recorded in response to electrical stimulation of the central end of the vagus nerve (Fig. 1*D*,*E*). The responses were observed in the area adjacent to the fourth ventricle and rostral from the calamus scriptorius (Fig. 1*D*; Movie 1), indicating that NTS astrocytes respond to vagal afferent inputs with rapid increases in intracellular [Ca^2+^].

Movie 1. Representative recording of NTS astrocytic [Ca^2+^]_i_ responses induced by VNS under the control conditions. 10.1523/JNEUROSCI.1438-19.2020.video.1

### Brainstem astrocytes express 5-HT_2A_ receptors

**Figure 2.**
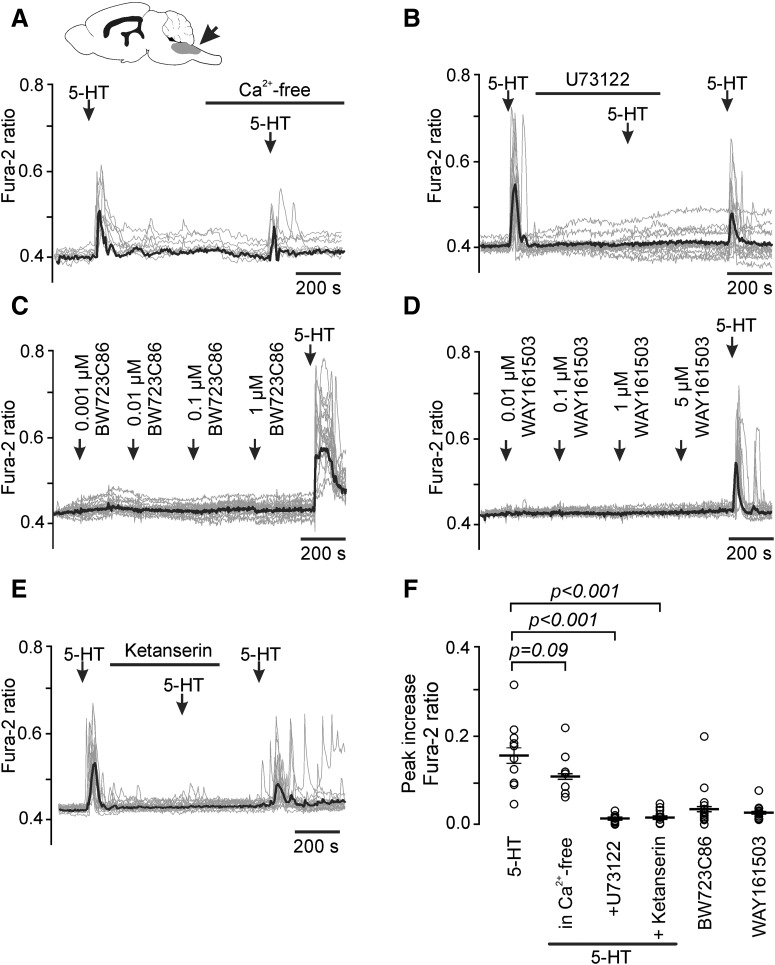
Brainstem astrocytes in culture respond to 5-HT with increases in [Ca^2+^]_i_ via activation of 5-HT_2A_ receptors. ***A***, Brainstem astrocytes respond to 5-HT (10 μm) with elevations in [Ca^2+^]_i_. 5-HT-induced [Ca^2+^]_i_ responses are independent of extracellular Ca^2+^. ***B***, 5-HT-induced [Ca^2+^]_i_ responses are blocked by the PLC-inhibitor U73122 (5 μm). ***C***, Brainstem astrocytes do not respond to 5-HT_2B_ receptor agonist BW723C86 and (***D***) 5-HT_2c_ receptor agonist WAY161503. ***E***, 5-HT-induced [Ca^2+^]_i_ responses are blocked by the 5-HT_2A_ receptor antagonist ketanserin (0.01 μm). ***F***, Summary data illustrating peak [Ca^2+^]_i_ responses in the brainstem astrocytes induced by 5-HT, 5-HT receptor agonists (BW723C86, 1 μm; WAY161503, 5 μm) and 5-HT in Ca^2+^-free conditions, or in the presence of U73122 or ketanserin (Student's *t* test).

As there is evidence that 5-HT is co-released with glutamate from vagal afferents in the NTS (Ramage and Villalón, 2008), we tested for the presence of 5-HT receptors in brainstem astrocytes. Cultured brainstem astrocytes responded to application of 5-HT (10 μm) with profound elevations in intracellular [Ca^2+^] (0.164 ± 0.022 fura-2 ratio above the baseline, *n* = 10; Fig. 2*A*,*F*). 5-HT-induced Ca^2+^ responses were not affected in the absence of extracellular Ca^2+^ (Ca^2+^-free medium with the addition of 0.5 mm EGTA; 0.115 ± 0.022, *n* = 10, *t* test, *p* = 0.09; Fig. 2*A*,*F*), suggesting that 5-HT recruits Ca^2+^ from the intracellular stores, likely via activation of the G_q_-coupled 5-HT_2_ receptors (Hoyer et al., 2002). Indeed, [Ca^2+^]_i_ responses triggered by 5-HT in the brainstem astrocytes were abolished in the presence of phospholipase C inhibitor U73122 (5 μm; 0.011 ± 0.002, *n* = 15, *t* test, *p* < 0.001; Fig. 2*B*,*F*) and 5-HT_2A_ receptor antagonist ketanserin (0.01 μm; 0.015 ± 0.004, *n* = 16, *t* test, *p* < 0.001; Fig. 2*E*,*F*). Neither 5-HT_2B_ agonist BW723C86 (in concentrations 0.001–1 μm) nor 5-HT_2C_ agonist WAY161503 (in concentrations 0.01–5 μm) had an effect on [Ca^2+^]_i_ in brainstem astrocytes (Fig. 2*C*,*D*,*F*). These data indicate that responses of brainstem astrocytes to 5-HT are mediated by 5-HT_2A_ receptors.

**Figure 3.**
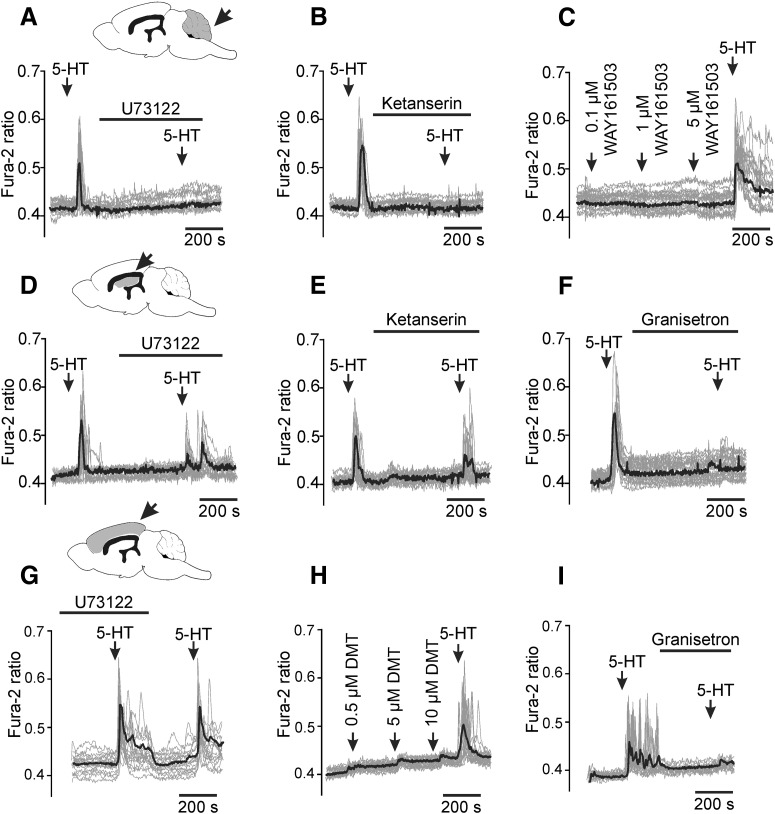
Distinct receptors mediate 5-HT-induced [Ca^2+^]_i_ responses in astrocytes residing in different parts of the CNS. Cerebellar astrocytes express 5-HT_2A_ receptors: (***A***) 5-HT (10 μm)-induced [Ca^2+^]_i_ responses in cultured cerebellar astrocytes are blocked by U73122 (5 μm) or (***B***) 5-HT_2A_ receptor antagonist ketanserin (0.01 μm). ***C***, Cerebellar astrocytes do not respond to 5-HT_2c_ receptor agonist WAY161503. Hippocampal astrocytes express 5-HT_3_ receptors: (***D***) 5-HT-evoked [Ca^2+^]_i_ responses in hippocampal astrocytes are unaffected by U73122 (10 μm) or (***E***) ketanserin (0.01 μm) but are blocked by (***F***) 5-HT_3_ receptor antagonist granisetron (20 μm). Cortical astrocytes express 5-HT_3_ receptors: (***G***) 5-HT-evoked [Ca^2+^]_i_ responses in cortical astrocytes are unaffected by U73122 (10 μm) and do not respond to (***H***) 5-HT_2_ receptor agonist DMT (0.5–10 μm). ***I***, 5-HT-evoked [Ca^2+^]_i_ responses in cortical astrocytes are blocked by granisetron (20 μm).

For comparison, we analyzed [Ca^2+^]_i_ responses induced by 5-HT in astrocytes residing in other areas of the brain (cerebellum, hippocampus and cortex; Fig. 3). The results obtained suggest that the profile of 5-HT receptors expressed by brainstem astrocytes is distinct from that of the forebrain astrocytes. Similarly to the brainstem astrocytes, [Ca^2+^]_i_ responses induced by 5-HT in cerebellar astrocytes (Bergmann glia) were blocked by U73122 or ketanserin (Fig. 3*A*,*B*). The 5-HT_2C_ agonist WAY161503 had no effect on Bergmann glia (Fig. 3*C*), suggesting that cerebellar astrocytes express 5-HT_2A_ receptors. In contrast, [Ca^2+^]_i_ responses induced by 5-HT in hippocampal and cortical astrocytes were not affected by U73122 (Fig. 3*D*,*G*) or ketanserin (Fig. 3*E*), but were abolished in the presence of the 5-HT_3_ antagonist granisetron (Fig. 3*F*,*I*). The 5-HT_2A/2C_ receptor agonist DMT had no effect on [Ca^2+^]_i_ in cortical astrocytes (Fig. 3*H*). These data suggest that, in contrast to the brainstem astrocytes, the forebrain astroglia express 5-HT_3_ receptors.

### 5-HT_2A_ receptors mediate [Ca^2+^]_i_ responses in NTS astrocytes evoked by vagus nerve simulation

**Figure 4.**
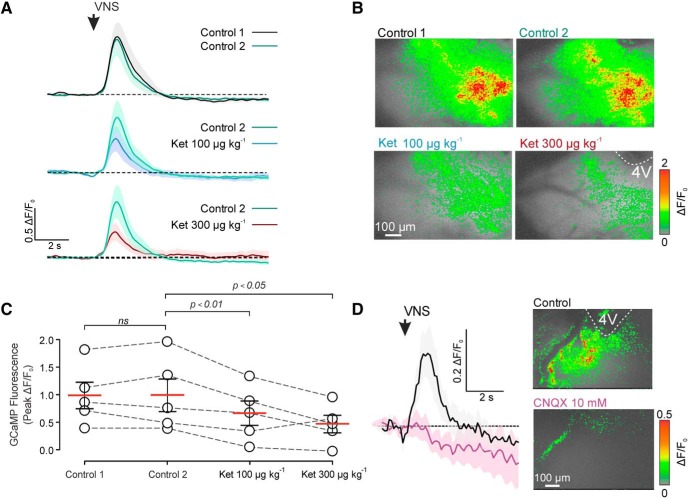
[Ca^2+^]_i_ responses in NTS astrocytes induced by VNS are mediated by 5-HT and glutamate. ***A***, Representative recordings illustrating changes in GCaMP6 fluorescence (±SEM) reporting [Ca^2+^]_i_ dynamics in response to VNS in the absence and presence of 5-HT_2A_ receptor antagonist ketanserin (Ket; 100 and 300 μg/kg, i.v.; *n* = 5). ***B***, Representative false color images of peak increases in GCaMP6 fluorescence induced by VNS in the absence and the presence of ketanserin. ***C***, Summary data illustrating the effect of ketanserin on peak [Ca^2+^]_i_ responses induced by VNS in the NTS astrocytes (one-way ANOVA followed by Sidak's multiple comparisons test). ns - not significant. ***D***, Representative recordings and false color images illustrating changes in GCaMP6 fluorescence in response to VNS in the absence and presence of an AMPA receptor antagonist CNQX (10 mm, topical application).

To determine whether 5-HT_2A_ receptors expressed by brainstem astrocytes are functional *in vivo*, we next studied the effect of 5-HT_2A_ receptor blockade on [Ca^2+^]_i_ responses of NTS astrocytes evoked by vagus nerve stimulation. Ketanserin dose-dependently decreased the amplitudes of [Ca^2+^]_i_ responses in NTS astrocytes evoked by stimulation of vagal afferents (Δ*F*/*F*_0_ = 0.65 ± 0.2 and 0.46 ± 0.16 from a baseline of 1.0 ± 0.3 Δ*F*/*F*_0_, following administration intravenously in doses of 100 and 300 μg/kg, respectively; *n* = 5, one-way ANOVA, *p* < 0.01; Fig. 4*A–C*; Movies 1, 2). In similar experimental conditions, [Ca^2+^]_i_ responses in NTS astrocytes evoked by vagus nerve simulation were abolished by AMPA receptor blockade with CNQX (10 mm; applied topically to the dorsal brainstem; Fig. 4*D*).

Movie 2. Representative recording of NTS astrocytic [Ca^2+^]_i_ responses induced by VNS 10 min after the application of 300 μg/kg ketanserin (i.v.). 10.1523/JNEUROSCI.1438-19.2020.video.2

### Blockade of vesicular release mechanisms in NTS astrocytes increases the baroreflex sensitivity

**Figure 5.**
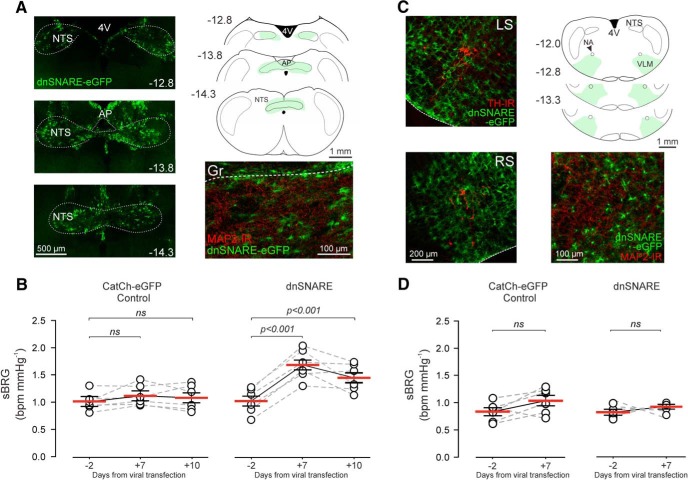
dnSNARE expression in NTS astrocytes increases the baroreflex sensitivity. ***A***, Photomicrographs of the coronal sections of the rat brainstem illustrating the expression of dnSNARE in astrocytes of the NTS and wider dorsal vagal complex. Astrocytes expressing the transgene were identified by eGFP immunofluorescence. Schematic drawings illustrate the spatial extent of dnSNARE expression. Distance from bregma (in mm) is indicated. Higher magnification image of dnSNARE-eGFP expression (green) in astrocytes of the intermediate NTS shows no colocalization of expression with MAP2-immunoreactivity (red). Gr, gracile nucleus. ***B***, Summary data illustrating daytime values of sBRG in conscious freely-moving animals transduced to express the control transgene (CatCh-eGFP) or dnSNARE in the NTS astrocytes (one-way ANOVA). ***C***, Photomicrographs of the coronal sections of the rat brainstem illustrating the expression of dnSNARE in astrocytes of the ventrolateral medulla (VLM). Pre-sympathetic neurons of the VLM are identified by TH-immunoreactivity. Schematic drawings illustrate the spatial extent of dnSNARE expression in the VLM region. Distance from bregma (in mm) is indicated. Higher-magnification image of dnSNARE-eGFP expression (green) in the VLM shows no colocalization of expression with MAP2-immunoreactivity (red). LS, Left side; RS, right side; NA, nucleus ambiguus. ***D***, Summary data illustrating daytime values of sBRG in conscious freely-moving animals transduced to express CatCh-eGFP or dnSNARE in astrocytes of the VLM (one-way ANOVA). ns - not significant.

To determine the functional significance of the recorded astroglial Ca^2+^ responses, we next determined whether blockade of Ca^2+^-dependent vesicular release mechanisms in the NTS astrocytes has an effect on baroreflex. In conscious freely moving rats, dnSNARE expression in astrocytes of the NTS (Fig. 5*A*) led to a significant increase in baroreflex sensitivity, when assessed 7 and 10 d after the injections of the viral vectors when the expression of the transgene peaks (sBRG 1.7 ± 0.11 and 1.5 ± 0.10 vs 1.0 ± 0.10 bpm/mmHg at baseline, *p* < 0.001, one-way ANOVA; Fig. 5*B*). Baroreflex sensitivity was unaffected in animals transduced to express the control transgene in the NTS astrocytes (sBRG 1.1 ± 0.08 and 1.1 ± 0.13 vs 1.0 ± 0.07 bpm/mmHg at baseline, *p* < 0.05, one-way ANOVA; Fig. 5*B*). Expression of dnSNARE or control transgene in astrocytes of the VLM (Fig. 5*C*) had no effect on baroreflex sensitivity (Fig. 5*D*).

### P2Y_1_ receptors in the NTS modulate the baroreflex

**Figure 6.**
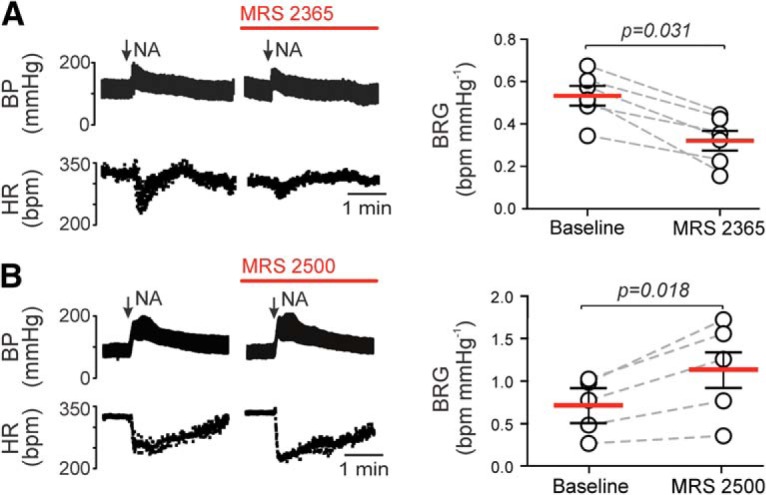
P2Y_1_ receptors in the NTS modulate baroreflex sensitivity. ***A***, P2Y_1_ receptor agonist MRS 2365 (100 μm, topical application) inhibits bradycardia induced by baroreceptor activation with systemic norepinephrine (NA; 0.1 μg/kg, i.v.) in anesthetized rats (Wilcoxon matched-pairs signed rank test). ***B***, P2Y_1_ receptor antagonist MRS 2500 (5 μm, topical application) potentiates the bradycardia induced by baroreceptor activation with NA (0.1 μg/kg, i.v.) in anesthetized rats (Wilcoxon matched-pairs signed rank test). BP, Arterial blood pressure; HR, heart rate. Representative responses recorded before and 15 min after each drug application are shown.

ATP is one of the main signaling molecules releases by astrocytes in response to elevations in [Ca^2+^]_i_ (Gourine and Kasparov, 2011). We next hypothesized that ATP is released by astrocytes in response to incoming afferent activity and acts on P2Y_1_ receptors expressed by NTS inhibitory interneurons to restrain the expression of baroreflex. An analogous mechanism involving ATP-induced P2Y_1_ receptor-mediated activation of inhibitory interneurons has been described in the cortex (Wang et al., 2012). Baroreflex sensitivity was assessed in animals anesthetized with α-chloralose before and after application of a potent and selective P2Y_1_ receptor agonist MRS 2365 (100 μm) or P2Y_1_ receptor antagonist MRS 2500 (5 μm; Kim et al., 2003) topically to the dorsal brainstem. Baroreflex was activated by bolus injections of norepinephrine. Activation of P2Y_1_ receptors with MRS 2365 reduced the baroreflex gain (0.3 ± 0.05 vs 0.5 ± 0.05 bpm/mmHg at baseline, *p* = 0.031, Wilcoxon matched-pairs signed rank test; Fig. 6*A*), whereas blockade of P2Y_1_ receptors with MRS 2500 increased the baroreflex gain (1.1 ± 0.26 vs 0.7 ± 0.15 bpm/mmHg at baseline, *p* = 0.018, Wilcoxon matched-pairs signed rank test; Fig. 6*A*).

## Discussion

The importance of astrocytes in supporting the function of NTS circuitry has been suggested previously by (Lin et al., 2013), who reported that ablation of NTS astrocytes using the toxin saporin leads to cardiovascular reflex inhibition, lability of arterial pressure, damage of cardiac myocytes and, in some animals, sudden cardiac death. Considering the important role played by astrocytes in providing structural and metabolic support, as well as K^+^ buffering and glutamate recycling, it is not surprising that ablation of astrocytes has a major impact on the neuronal function and, perhaps, nerve cell viability. Therefore, the role of astrocytes in the subtleties of neuronal processing and integration of afferent information within the NTS remain unknown. In this study we aimed to determine the role of astrocytes in the NTS mechanisms that mediate the baroreceptor reflex pathway.

*In vivo* calcium imaging demonstrated that NTS astrocytes respond to vagal afferent input with increases in intracellular [Ca^2+^]. These data are in agreement with the observations by McDougal et al. (2011) who reported that NTS astrocytes respond with increases in [Ca^2+^]_i_ to stimulation of the solitary tract in slices (McDougal et al., 2011). [Ca^2+^]_i_ responses in NTS astrocytes induced by vagus nerve stimulation were reduced or abolished by either 5-HT_2A_ or AMPA receptor blockade. This is consistent with the evidence that 5-HT is a co-transmitter released by vagal afferent terminals (Thor and Helke, 1989).

5-HT receptors have been shown to be expressed by astrocytes in many brain areas (Sandén et al., 2000), but have not been identified in the brainstem astroglia. Pharmacological analysis of 5-HT-induced [Ca^2+^]_i_ responses in cultured astrocytes indicated that brainstem astrocytes express 5-HT_2A_ receptors. Although 5-HT_3_ receptors have been previously suggested to be expressed by NTS astrocytes (Huang et al., 2004), we found no evidence for their involvement in mediating the actions of 5-HT in brainstem astroglia. NTS astrocytes appear to be distinct from the forebrain astrocytes (cortical and hippocampal) where 5-HT effects are mediated solely by ionotropic 5-HT_3_ receptor activation.

The data obtained in this study show that 5-HT_2A_ receptors partially mediate [Ca^2+^]_i_ responses in NTS astrocytes evoked by vagus nerve stimulation as these were reduced by ∼50% in the presence of the 5-HT_2A_ antagonist ketanserin. However, 5-HT released as a result of enhanced vagal afferent activity alone was unable to trigger significant increases in astrocytic [Ca^2+^]_i_, as blockade of AMPA receptors completely abolished these responses. It is important to note that vagal afferents are not the only source of 5-HT in the NTS (Hosford et al., 2015). 5-HT-containing neurons of the brainstem raphe send projections to the NTS and could also be activated by reciprocal projections from the NTS (Thor and Helke, 1989; Rosin et al., 2006).

In an experiment involving specific blockade of Ca^2+^-dependent vesicular release mechanisms in the NTS astrocytes (by dnSNARE expression), we determined the functional significance of astroglial signaling in operation of the key homeostatic reflex—the baroreceptor reflex. It was found that inhibition of Ca^2+^-dependent astroglial signaling increased the baroreflex sensitivity when assessed in awake behaving rats. To determine whether this effect is attributed specifically to the NTS astrocytes, we also targeted the regions of the ventrolateral medulla that harbor both pre-sympathetic circuits (Marina et al., 2011) and cardiac vagal preganglionic neurons of the nucleus ambiguus (Gourine et al., 2016), both critically important for the operation of the baroreflex. Interestingly, despite widespread dnSNARE expression in astrocytes of the VLM, no effect on spontaneous baroreflex gain was detected, indicating a very specific role for NTS astrocytes in operation of this key cardiovascular reflex.

Our conclusions drawn from the data obtained in the experiments involving viral gene transfer in brainstem astrocytes rely on the enhanced GFAP promoter specificity. This vector system has been validated and demonstrated to be highly specific in targeting astroglial cells (Gourine et al., 2010; Rajani et al., 2018; Sheikhbahaei et al., 2018b), albeit in other areas of the brainstem. Additional verification of the expression specificity in the NTS showed that transgene expression is confined to non-neuronal cells, as no cells expressing eGFP (the reporter gene used in both viral vectors) showed MAP2 immunoreactivity. Further, expression of the viral vectors and the actions of the pharmacological agents used in this study are not confined to the NTS, but rather to the dorsal vagal complex. There is strong P2Y_1_ receptor presence throughout the dorsal vagal complex, including the area postrema (Fong et al., 2002), the area shown to modulate the baroreflex via its projections to the NTS (Shapiro and Miselis, 1985; Johnson and Gross, 1993). However, the area postrema does not seem to be directly involved in the baroreflex pathway, but can modulate NTS circuit activity by responding to various circulating factors, as it is positioned outside of the blood–brain barrier (Tan et al., 2007). Additionally, the dorsal vagal motor nucleus may modulate the baroreflex, but only in the pathophysiological context, such as in conditions of systemic arterial hypertension (Moreira et al., 2018). Together, the data obtained in the present study strongly suggest that the NTS is the dorsal brainstem site where the altered astroglial function has a major impact on baroreflex sensitivity.

One of the main astroglial signaling molecules is recognized to be ATP, which is known to inhibit local neuronal activity indirectly following rapid breakdown to adenosine—a mechanism first reported to operate in retina (Newman, 2003). Indeed, activation of adenosine A_1_ receptors in the NTS inhibits baroreflex sensitivity (Scislo and O'Leary, 2005). However, the data obtained in this study suggest the existence of a different mechanism, which is independent of adenosine actions. Pharmacological inhibition of P2Y_1_ receptors was found to have a similar effect on baroreflex as blockade of Ca^2+^-dependent vesicular release in NTS astrocytes expressing dnSNARE. These data suggest that, upon activation by afferent input, the NTS astrocytes release ATP which acts on NTS inhibitory neurons expressing P2Y_1_ receptors, a mechanism analogous to that described by Wang et al. (2012) in the cortex. It is also worth noting that ADP is the more potent ligand at the P2Y_1_ receptors (Waldo and Harden, 2004), therefore, the signaling pathway proposed could require (or be potentiated by) breakdown of ATP to ADP by ectonucleotidases encountered in the extracellular space.

Baroreceptor reflex is critically important for the short term (beat-to-beat) control of the arterial blood pressure. There is strong evidence that impaired baroreflex function contributes to the development of cardiovascular disease and serves as a robust predictor of cardiovascular and all-cause mortality (La Rovere et al., 1998, 2001; McCrory et al., 2016). The mechanisms underlying impairment of baroreflex function in pathological conditions remain largely unknown. Previously proposed mechanisms may involve activation of the cardiac sympathetic afferent reflex, which alters the baroreflex sensitivity via angiotensin II type 1 receptors in the NTS (Kasparov and Paton, 1999; Gao et al., 2005) and/or reduction of brain-derived neurotropic factor actions in the NTS (Becker et al., 2016). The results of the present study offer another plausible mechanism. Various pathological conditions that are associated with the development of the systemic and central inflammatory response leading to activation of the NTS glia (astroglia and microglia) would be expected to facilitate the release of ATP, increase the concentration of ATP/ADP in the NTS extracellular milieu and inhibit the baroreflex centrally. Indeed, activation of astrocytes and reactive astrogliosis have been reported after the CNS trauma, infection, ischemia, stroke, and in autoimmune disease (Sofroniew and Vinters, 2010). Higher levels of “ambient” ATP/ADP released by activated astrocytes and microglia would be expected to reduce the baroreflex sensitivity via P2Y_1_ receptor-mediated NTS mechanism described here.

Additionally, repeated activation of chemosensory inputs had been shown to inhibit the baroreflex and is thought to contribute to the pathology of various conditions including sleep apnea (Mifflin et al., 2015). Activation of chemosensory inputs increases extracellular 5-HT concentration in the NTS via release from afferent terminals, and also by the inputs from the central chemosensory sites (Kellett et al., 2005; Wu et al., 2019). This would be expected to maintain “activation” of astrocytes and decrease the baroreflex sensitivity. Previous studies of the role of 5-HT_2_ receptors within the NTS suggested that the astrocytic 5-HT_2A_ pathway is unlikely to be active under normal physiological conditions. Indeed, there is evidence that 5-HT_2A_ receptor blockade in the NTS did not alter the baroreflex sensitivity (Sévoz-Couche et al., 2006; Comet et al., 2007). However, these studies also reported a facilitatory effect of 5-HT_2A_ receptor activation on baroreflex. It is possible that the 5-HT_2A_ receptors expressed by astrocytes are recruited primarily in pathophysiological conditions (eg. sleep apnoea) and, only in these circumstances, modify the baroreflex via astrocytic release of ATP.

**Figure 7.**
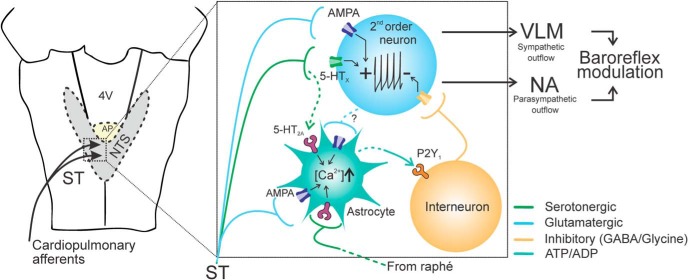
Schematic illustration of the proposed NTS mechanisms involved in modulation of the baroreflex. Vagal afferent terminals release 5-HT and glutamate acting on second order relay neurons and astrocytes in the NTS. In response to incoming afferent information, NTS astrocytes release ATP which restricts the expression of baroreflex via activation of P2Y_1_ receptors on NTS inhibitory interneurons. ST, Solitary tract; NA, nucleus ambiguus; VLM, ventrolateral medulla.

In conclusion, the data obtained in the present study suggest that astrocytes are integral components of the NTS mechanisms that process incoming afferent information. NTS astrocytes are activated by glutamate (McDougal et al., 2011) and 5-HT released by vagal afferent fibers and acting at AMPA and 5-HT_2A_ receptors, respectively (Fig. 7). Activation of astrocytes in response to afferent stimulation leads to the release of ATP acting on P2Y_1_ receptors to modulate the baroreflex sensitivity. Together, these results add to the growing body of evidence supporting an active role of astrocytes in the information processing in the CNS.
